# Advances in Vertebrate (Cyto)Genomics Shed New Light on Fish Compositional Genome Evolution

**DOI:** 10.3390/genes14020244

**Published:** 2023-01-17

**Authors:** Dominik Matoulek, Bruno Ježek, Marta Vohnoutová, Radka Symonová

**Affiliations:** 1 Department of Physics, Faculty of Science, University of Hradec Králové, 500 03 Hradec Králové, Czech Republic; 2 Faculty of Informatics and Management, University of Hradec Králové, Rokitanského 62, 500 02 Hradec Králové, Czech Republic; 3 Department of Computer Science, Faculty of Science, University of South Bohemia, Branišovská 1760, 370 05 České Budějovice, Czech Republic; 4 Department of Bioinformatics, Wissenschaftszentrum Weihenstephan, Technische Universität München, 85354 Freising, Germany; 5 Institute of Hydrobiology, Biology Centre of the Czech Academy of Sciences, 370 05 České Budějovice, Czech Republic

**Keywords:** compositional cytogenomics, AT/GC evolution, GC content, genome evolution, GC landscape pipeline

## Abstract

Cytogenetic and compositional studies considered fish genomes rather poor in guanine-cytosine content (GC%) because of a putative “sharp increase in genic GC% during the evolution of higher vertebrates”. However, the available genomic data have not been exploited to confirm this viewpoint. In contrast, further misunderstandings in GC%, mostly of fish genomes, originated from a misapprehension of the current flood of data. Utilizing public databases, we calculated the GC% in animal genomes of three different, technically well-established fractions: DNA (entire genome), cDNA (complementary DNA), and cds (exons). Our results across chordates help set borders of GC% values that are still incorrect in literature and show: (i) fish in their immense diversity possess comparably GC-rich (or even GC-richer) genomes as higher vertebrates, and fish exons are GC-enriched among vertebrates; (ii) animal genomes generally show a GC-enrichment from the DNA, over cDNA, to the cds level (i.e., not only the higher vertebrates); (iii) fish and invertebrates show a broad(er) inter-quartile range in GC%, while avian and mammalian genomes are more constrained in their GC%. These results indicate no sharp increase in the GC% of genes during the transition to higher vertebrates, as stated and numerously repeated before. We present our results in 2D and 3D space to explore the compositional genome landscape and prepared an online platform to explore the AT/GC compositional genome evolution.

## 1. Introduction

Fish genomes are traditionally considered GC-poor in comparison to mammalian and avian genomes, e.g., [1]. This is ascribed to a hypothesized abrupt increase in the GC% of particularly genic regions during the evolution of birds and mammals and its conservation in these vertebrates [2]. This deeply anchored opinion originates from only a few studies on the AT/GC composition of genomes in warm-blooded vertebrates in contrast to cold-blooded vertebrates [3]. Other studies broadly generalized their analyses of a very limited number of species or even of a single fish species [4,5,6,7]. On the other hand, there is strong support from the field of traditional as well as molecular cytogenetics showing the absence of the G-banding or AT- and GC-specific fluorescence staining patterns in fish, e.g., in [8,9], which is only a qualitative visualization on the chromosome. This absence of any banding pattern in fish and its existence in mammals and birds still needs to be clarified. In the meantime, an increasing amount of genomic data became publicly available to better explore the compositional genome evolution of animals in databases such as the NCBI/Genome [10] or Ensembl [11]. This wealth of data, however, has not yet been exploited since one line of the AT/GC genome evolution research focuses on a recombination-based GC-biased gene conversion in coding regions, particularly in mammals, e.g., [12]. Another line of research ceased with the publication of several graphs of isochore families in fish [13], stating that fish genomes are GC-poor and AT/GC homogeneous. This was proved false by evidence of the mammalian type of the AT/GC genome heterogeneity in an ancient lineage of ray-finned fish called gars [9] using both cytogenetics and bioinformatics. This finding initiated a quantitative approach to genome composition, regarding not only chromosome counts, size, and morphology as intrinsic drivers of the AT/GC genome evolution [14]. Transposable elements (TEs) were also shown as major and so far omitted players in this process since their proportion correlates with the host genome size in fish and their GC% correlates with the GC% of the host genome in fish [15]. Moreover, TEs were proposed to homogenize GC% along chromosomes in fish when compared with mammals [16]. In parallel, the very existence of isochores was repeatedly proved false, e.g., [17]. This again opened up the question of the genome GC evolution in animals. In plants, the GC% has been successfully correlated with genomic and cellular traits and linked with adaptive consequences and evolutionary history [18,19]. A recent study shows that the GC% of plant genes is linked to past gene duplications [20]. Such knowledge is missing for animals, where it is not yet clearly elucidated whether the GC% has adaptive or (nearly) neutral evolutionary consequences [2].

Hence, the next step is to explore the AT/GC organization in genome fractions such as genes and those with and without TEs to assess their role in genome composition together with the influence of other elements among animals. To do so, high-quality animal genome assemblies deposited in the public database Ensemble [11] are suitable since three relevant fractions are available for numerous species, i.e., the entire genome DNA, the complementary DNA (cDNA), and the coding sequences (cds, exons). These three genome fractions represent technically well-established different phases of storage and processing of genetic information with different proportions of TEs: i. the entire genome DNA includes genic, intergenic, repetitive (i.e., also TEs), and regulatory regions; ii. the cDNA fraction is related to regulation and further tuning of transcription and includes untranslated regions (UTRs), the 5′-UTR, i.e., the region upstream of the start codon [21], and the 3’-UTR, i.e., the region downstream of the stop codon [22], and introns. Both UTRs harbor TEs [6]. Introns are known as targets of TE insertions in lower eukaryotes [23], fish [24], and mammals [25]. Finally, iii. the cds fraction, i.e., the coding sequences or coding regions [26], here interchangeable with exons/exome, are translated into amino acids according to the genetic code. Exons are considered mostly devoid of TEs, although some cds arose from the exonization of TEs [25]. Then, they become regular exons and not TEs anymore.

The main aims of this study were to assess the AT/GC compositional cytogenomic organization in animals by exploitation of the currently available genome assemblies and related cDNA and cds sequences. Our results bring novel insights into the similarities and differences between the genomes of cold- and warm-blooded animals to a so far unprecedented extent, and above all unbiased by former approaches. This study belongs to a long-term effort to elucidate the AT/GC compositional evolution across invertebrates and, finally, vertebrates, where so far ununderstood differences exist between fish (generally cold-blooded vertebrates) and higher (warm-blooded) vertebrates.

## 2. Results

**Figure 1:**
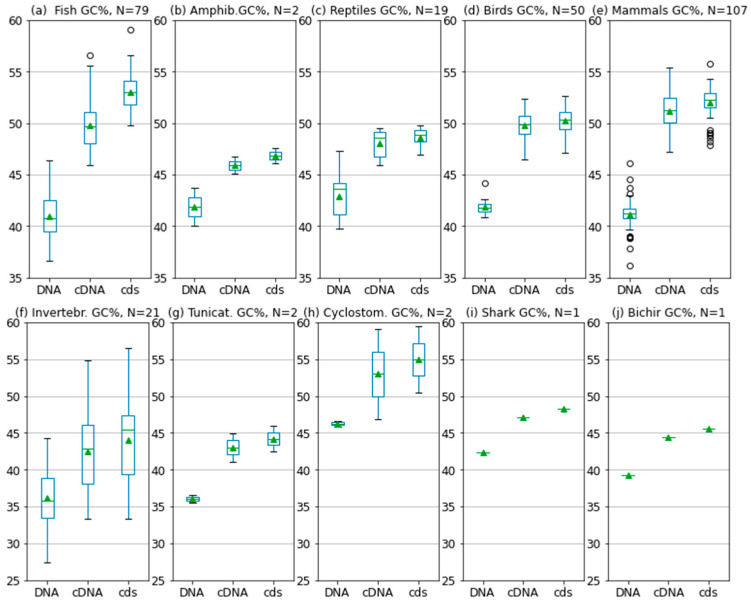
Overview of the GC% values (y axis) of the three basic genome fractions in vertebrates (**a**–**e**,**h**–**j**) and selected invertebrates (**f**,**g**) utilizing available relevant FASTA sequences from Ensembl release 108 and from Ensembl Metazoa [11].

**Figure A1:**
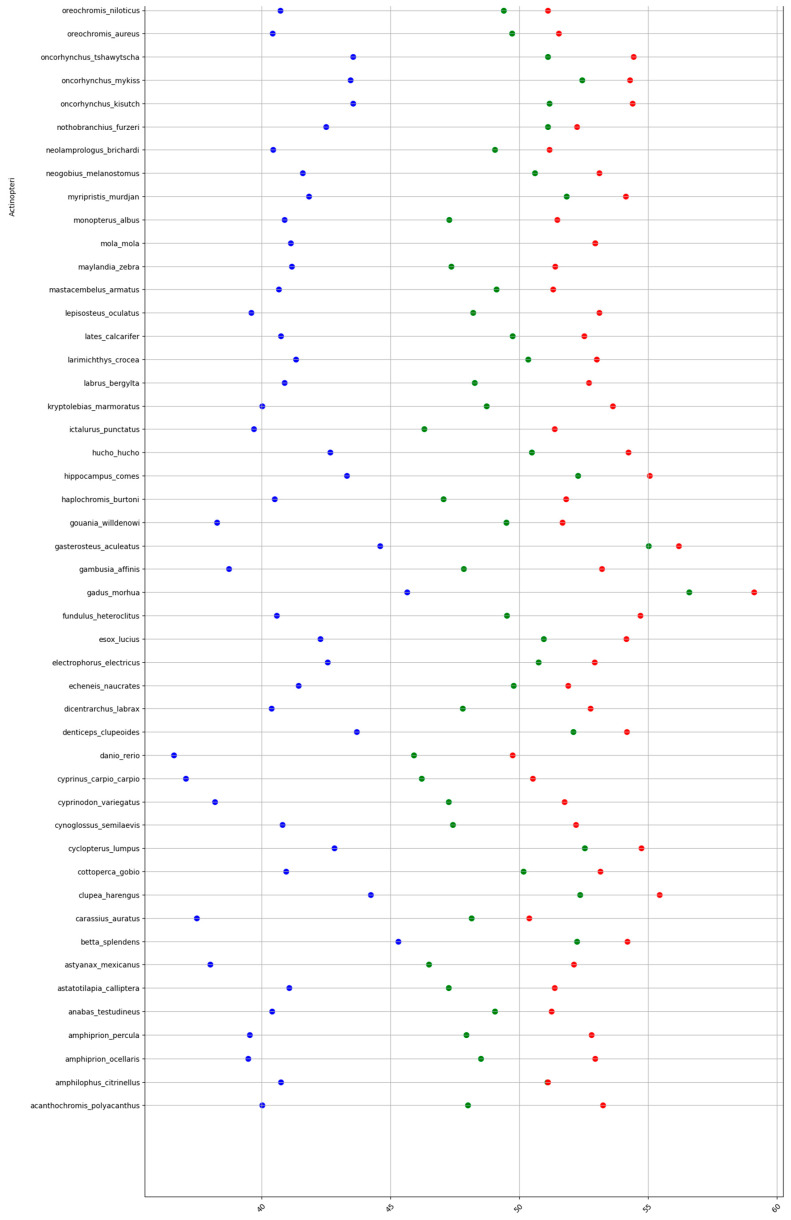
GC% in fish at the levels of DNA (blue), cDNA (green), and cds (red). A partial table shown here. The complete dataset is too large and is available at github.com/martavohnoutova.

**Figure A2:**
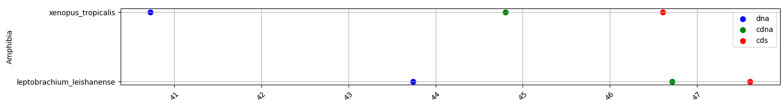
GC% in amphibians at the levels of DNA (blue), cDNA (green), and cds (red).

**Figure A3:**
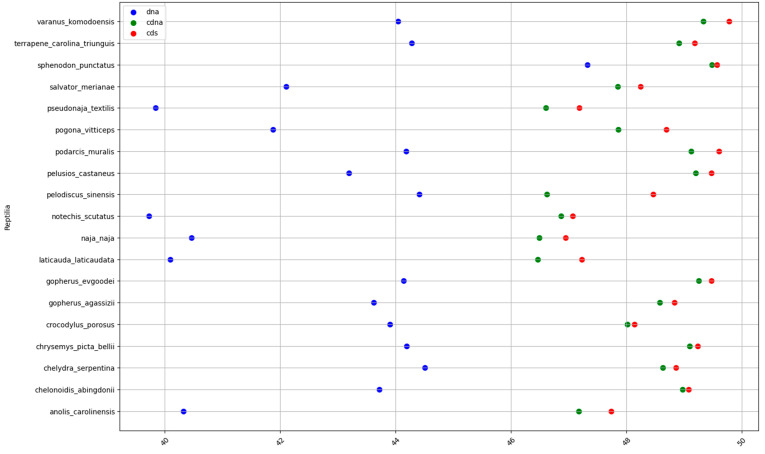
GC% in reptiles at the levels of DNA (blue), cDNA (green), and cds (red).

**Figure A4:**
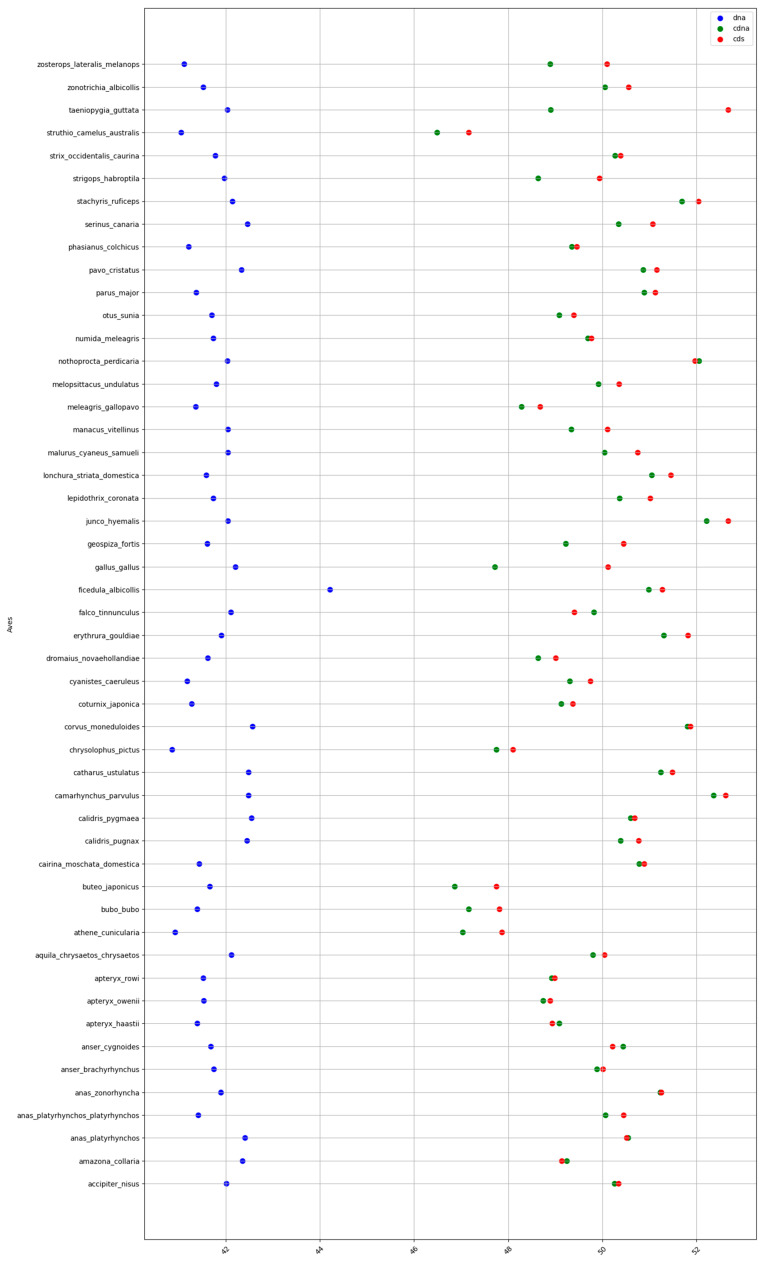
GC% in birds at the levels of DNA (blue), cDNA (green), and cds (red).

**Figure A5:**
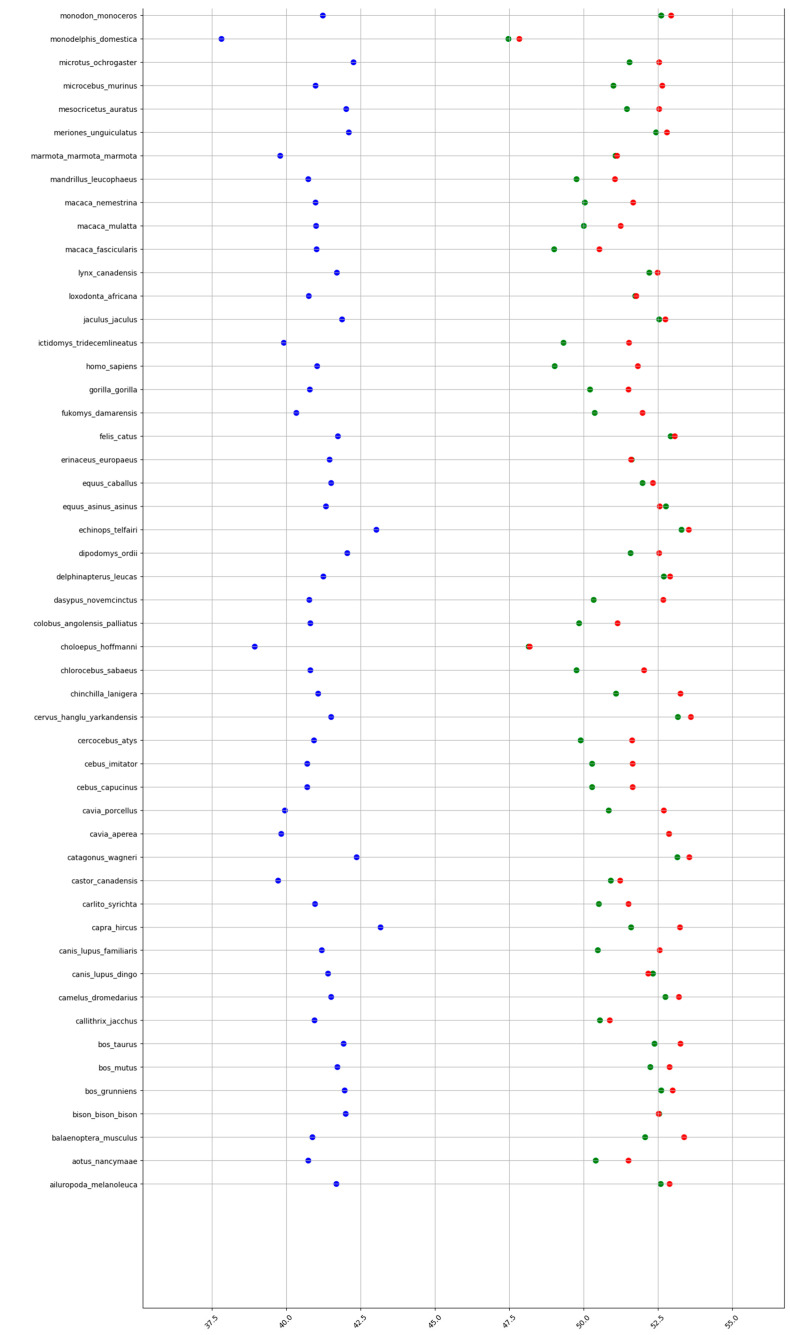
GC% in mammals at the levels of DNA (blue), cDNA (green), and cds (red). A partial table shown here. The complete dataset is too large and is available at github.com/martavohnoutova.

Our results on the three genome fractions and their GC% across animals are provided in a 2D and 3D way. The 2D presentation serves as a quick overview and summarization (boxplots in Figure 1 and tables in Figure A1, Figure A2, Figure A3, Figure A4 and Figure A5) of the respective values. The 3D visualization in a tailored graphic application presents values for the three genome fractions in the 3D space, which is a more natural way to deal with the three parameters. The 3D presentation visualizes the phylogenetic relationships of the species analyzed and a species-specific position on the AT/GC compositional landscape.

### 2.1. GC content of the Fractions DNA, cDNA, and cds in Animal Genomes

Fish and invertebrates (Figure 1a,f) showed a higher inter-quartile range (IQR) in the GC% of all DNA fractions than birds and mammals (Figure 1d,e). The mean values for each group and DNA fraction are in Table 1. The GC% of DNA and cds overlaps in fish and mammals. Since GC% of cDNA is slightly lower in fish than in mammals, the resulting difference between GC% of cDNA and cds is in fish larger than in mammals. On the other hand, fish reach a higher GC% of cds (52.5%) than both birds (50.1%, Figure 1d) and mammals (51.9%, Figure 1e). The two amphibians available, the Leishan spiny toad (*Leptobrachium leishanense*) and the tropical clawed frog (*Xenopus tropicalis),* show a comparable GC% of DNA (Figure 1b), but lower GC% of cDNA and cds when compared with fish and other vertebrates. For more amphibian species, the GC% of the entire DNA can be retrieved from the NCBI/Genome (Table 2). Reptiles show a higher IQR for the GC% of DNA (Figure 1c) than amphibians and intermediate GC% of cDNA and cds between amphibians and the higher vertebrates. The overall GC-richness of all genome fractions is the highest in the sea lamprey (DNA 45.8%, cDNA 59.1%, and cds 59.4%). The sea lamprey genome increases both the IQR as well as the GC% for the group of cyclostomes. Hence, if lampreys were treated separately, the IQR would not reach such a broad value (Figure 1h). The highest GC% of the cds in the cyclostomes is interesting because cds is assumed to be devoid of repeats that otherwise occupy large fractions of genomes [27,28]. Ancestral tunicates are the GC-poorest among chordates (Figure 1g).

The lowest GC% together with the highest IQR was recorded in the small outgroup sample of invertebrates (Figure 1f). Their GC% overlap with those of the two tunicates (Figure 1g) available in Ensembl.

**Figure 2:**
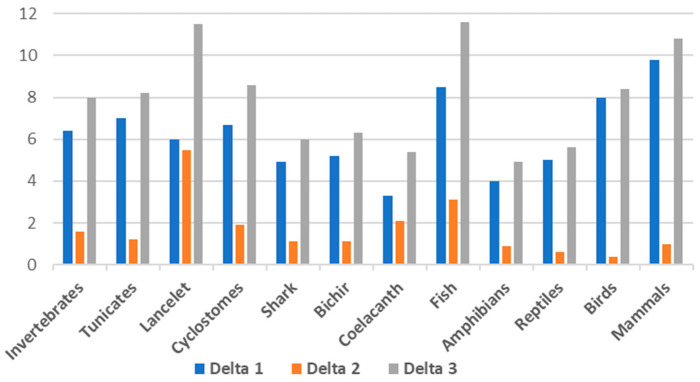
Visualization of the differences in GC% between the fractions DNA and cDNA (Delta 1), cDNA and cds (Delta 2), and between DNA and cds (Delta 3) as described in Table 1. Calculated from mean values for each group. One lancelet species is added (not present in Figure 1).

To assess the difference between each of the DNA fractions, we have calculated Delta 1 as the difference in GC% between the cDNA and DNA fractions, Delta 2 as the difference between the cds and DNA, and Delta 3 as the difference in GC% between the cds and DNA (Table 1, Figure 2). These results show that Delta 1, i.e., the difference in GC% between the entire genome and the cDNA, is the highest in mammals (Delta 1 = 9.8) followed by fish (Delta 1 = 8.5). The Delta 3, i.e., the difference between the cds and the entire genome, is in fish (Delta 3 = 11.6), lancelets (11.5), followed by mammals (10.8). The highest difference between the cds and cDNA is in the lancelet (Delta 2 = 5.5).

Our results have the potential to improve general knowledge of the intervals in the genome GC% in the main animal groups. To do so, we present an overview of the minimal and maximal verified GC% values in Table 2. This overview is intended as a reference for other authors since the seemingly simple GC% values have turned out to be problematic to deal with even in recent literature, e.g., [30,31].

### 2.2. 3D Visualization of GC% of DNA, cDNA and cds in Animal Genomes

**Figure 3:**
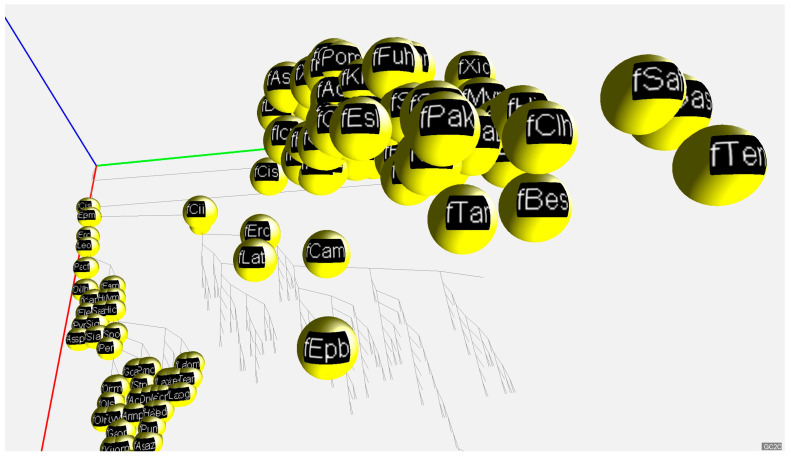
A 2D print screen of the graphic application GC2C visualizing GC% of DNA, cDNA and, cds fractions across animal lineages. The bottom part of the graph provides the phylogenetic relationships among the species. A function of the application moves the species to their specific position resulting from the GC% values of DNA, cDNA, and cds, respectively. Axes: red—GC% of DNA, green—GC% of cDNA, blue—GC% of cds. Here, fishes are visualized in details (yellow spheres).

**Figure 4:**
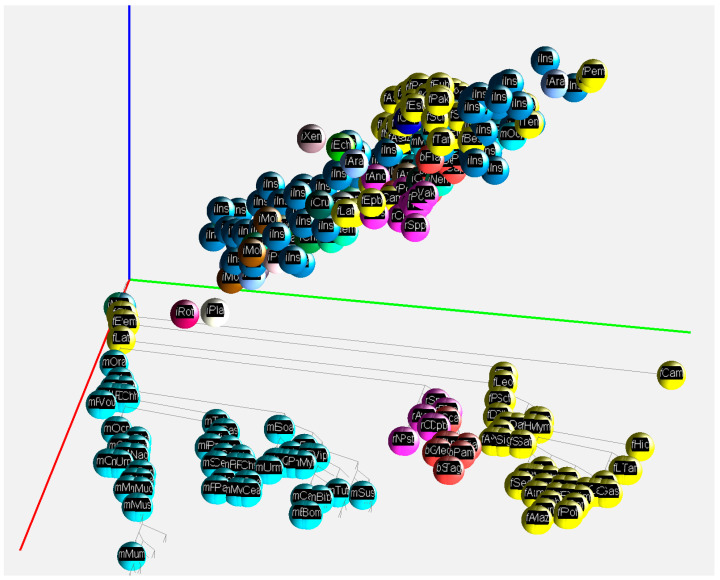
A 2D print screen of the application GC2C visualizing GC% of DNA, cDNA, and cds across animal lineages. A function of the application moves the species to their specific position resulting from the GC% values of DNA, cDNA, and cds, respectively. Axes: red—DNA, green—cDNA, blue—cds. Visualized fish (yellow), reptiles (violet), birds (red), mammals (turquoise), and insects (blue) as an outgroup.

For a convenient visualization of all three GC% values in the 3D space, we have developed a free application called GC2C. Each individual species is rendered as a small sphere and placed in 3D space. The position of the 3D sphere is based on a vector composed of GC% values for DNA, cDNA, and cds. Groups of species such as fish, mammals, birds, and others are colored the same. The species name, expressed by a short abbreviation, is mapped onto the surface of the sphere as a texture. The user can manipulate the 3D space by rotating and zooming the transformations. A phylogenetic tree is displayed in the bottom plane to better understand the relationships between the species. In both the 3D view and the bottom diagram, a user can select the sphere of each species and print detailed information. Only 2D print screens are shown here (Figure 3 and Figure 4).

This is a prototype version of GC2C based on currently available data. However, with the increasing availability of high(er)-quality genome assemblies, the 3D visualization tool will be populated with more precise data. The GC2C application is freely available on GitHub: https://github.com/fvbj/genomeVis.

## 3. Discussion

### 3.1. Animal Genome Composition and Outlines of Its Evolution

The GC% increase on the trajectory DNA-cDNA-cds was expected since different kinds of non-coding sequences are disregarded in the cDNA and finally, only the protein-coding regions remain in the cds. It is well known that exons are GC-rich, e.g., [32]. However, there is no sharp increase in the GC% of cds specific to avian and mammalian genomes, as previously stated [2] and repeated by others, e.g., [6]. Hence, the general GC-richness of cds across vertebrates can be considered as another indicator of the role of transposable elements (TEs) in shaping the overall AT/GC compositional landscape. TEs are known for their AT-richness, although not universal [33], and as AT-enriching factors, decreasing the GC% in large non-coding regions [16]. The more surprising is the fact that cDNA and cds of the basal lineage of cyclostomes represented by the sea lamprey are the GC-richest among vertebrates. Similarly, the higher differences in GC% between cDNA and cds in fish (Figure 1a and Figure 2, Table 1) when compared to other vertebrates might be ascribed to the GC-poor TEs residing in UTRs and introns. Their presence decreases the GC% of cDNA, and their removal from the cds brings the GC% of fish exons to comparable values as in mammals and birds.

### 3.2. Quality of Available Genome Assemblies Determines Our Possibilities to Analyse Data

We need to be aware that most of the currently available animal genomes, even the reference assemblies, are still incomplete with imperfections such as misassemblies and gaps [34,35]. Merely the latest assembly of the human genome, the Telomere-to-Telomere (T2T)-CHM13 version of the GRCh38, filled the remaining gaps for all chromosomes except Y and became the truly complete genome [36]. Such an effort cannot be expected, particularly in non-model species, at least in the near future. On the other hand, there are already initiatives combining short- and long-read sequencing [34] in non-model species (e.g., 16 species representing six major vertebrate lineages, including five fish and one skate species [37]), and even in large and complex species such as *Zea mays* or *Rana muscosa* [38]. This is a highly promising perspective since the quality of the starting data determines the quality of our results regardless of the availability and quality of the bioinformatics tools we have available, e.g., [16]. Another relevant and crucial aspect is that GC-rich regions were underrepresented in earlier assemblies (that are currently available), and that only the new and future-generated genomes will be truly representative for compositional studies [35,37]. This means that the new generation of assemblies will bring new and better opportunities to apply bioinformatics tools to resolve the issues of genome compositional evolution on the animal phylogenetic tree.

### 3.3. The Importance of Animal Genome Compositional Data

Several studies explored the animal genome organization and proportions of its major fractions with different goals. Firstly, the ratio of introns to intergenic sequence was found to be comparable across essentially all tested animals (68 species across 12 animal phyla, including some single-cell eukaryotes), with nearly all deviations dominated by increased intergenic sequence [39]. This ratio was utilized to assess the quality of gene annotations in the context of evolutionary studies and interpretations. The major finding is that genomes of model organisms have the ratio of introns to intergenic sequences much closer to 1:1, suggesting that the majority of published genomes of non-model organisms are under-annotated and consequently omit a substantial number of genes [39]. Here, again, the quality of genome assemblies plays a crucial role. Secondly, intron lengths, their counts, and GC% within genes affect the efficiency of pre-mRNA splicing and splice-site recognition [40]. The half-life and decay rates of mRNA transcripts are largely driven by the transcript GC% and length, determining the mRNA secondary structure [7,41]. Hence, these variables are crucial for other fields of biology besides compositional genome evolution; however, their scales are too fine to have the potential to explain the AT/GC heterogeneity in higher vertebrates. On the other hand, a comparative study of the exon-intron architecture found that the GC% of mammalian, avian, and frog exons negatively correlated with the length of their flanking introns [7,32]. In other species, including zebrafish and fugu, the opposite relationship was found [32]. Fish introns were found to be relatively short, highly variable, and with a bimodal size distribution [42]. An earlier study reported an ancient intron length expansion in the zebrafish lineage [43], showing that this species cannot represent fish genomes generally. Thirdly, several studies correlated regional GC% with the third codon position GC% (the GC3) of genes located in the region, e.g., [44]. However, these correlations were later found to be unsubstantiated [45] and hence abandoned. The only relevant study comparing the GC% of UTRs, introns, and cds in higher and lower vertebrates and invertebrates [6] included only zebrafish, together with humans, chickens, sea squirts, fruit flies, and worms. Therefore, its information value is largely limited and cannot be generalized.

### 3.4. Higher Constrains in cDNA and cds GC% in Higher Vertebrates

Potentially interesting findings are the less constraint values of GC% in all DNA fractions in fish and invertebrates, while more constraint in birds and mammals. These results might indicate a selective pressure on specific GC% values and their functionalities in higher vertebrates. This is supported by the fact that the GC% of coding and non-coding genic regions are strongly correlated and conserved among vertebrates [46]. On the other hand, the higher variation in the GC% among fish might result from their extreme diversity and evolutionary longevity and might have contributed to the distorted picture of the fish low-GC compositional landscape: (1) The most explored fish species, the zebrafish, is exceptionally GC-poor even among fishes. However, its genome was utilized frequently in comparative studies as “the typical fish genome” e.g., [4,5,6,7], because it was the first and best assembled fish genome for a long time; (2) the immense fish species diversity inevitably resulted in a highly biased and unrepresentative phylogenetic coverage of fish genomes analyzed. This might have easily led to biased results on the GC-richness of fish genomes, when mostly GC-poor or only moderately GC-rich fish genomes were explored. Even such a tremendous effort as the VGP produced in its initial phase high-quality genomes of six fish species [37]. Although insufficient to cover the fish species’ diversity now, this is promising for the future; finally, (3) non-teleost fish species, offering a window into the very deep vertebrates´ history and providing a link between teleost models and mammals, including humans, e.g., [47], are frequently omitted from large(r) scale studies. One opposite situation exists when only species of non-teleost fish lineages are presented and teleosts are omitted [46].

### 3.5. Delimiting Genome GC% Values of Invertebrates, Chordates and Higher Vertebrates

It has repeatedly turned out that it is not straightforward to set at least approximately correct limits of GC% across vertebrate genomes, particularly in fish. Clearly, incorrect values were published in peer-reviewed journals, e.g., GC% of DNA 31.5 % for the channel catfish by [31] or even more erroneous values of 25.4% for *Chionodraco hamatus* and 51.1% for *Squalius pyrenaicus* by [30]. Both these issues are explained in details and corrected in [48], however, no intervals of still acceptable values have been presented yet. Here, we wanted to contribute to setting the potential upper and lower bounds for the main groups (Table 2) utilizing manually curated datasets from NCBI/Genome [10]. The most complicated were the lower bounds for fish and reptiles, where numerous, largely incomplete scaffold-level genome assemblies prevail. For reptiles, following the instructions and rules in [48], we found a gap in the GC% values between 35% and 37%. From this value on, there was a gradual increase in GC% without any further gap. Hence, for the time being, we propose to set the lower-bound GC% value to 37% in *Notechis scutatus* with a chromosome-level assembly. It is, however, clear that with new genome assemblies available (i.e., both new species and improved versions of assemblies), these limits will be modified in the future.

## 4. Materials and Methods

We utilized the DNA, cDNA, and cds FASTA sequence data of all chordates provided by the latest release of Ensembl (108, published in October 2022) and Ensemble Metazoa Release 54 for selected insect species as an invertebrate outgroup [11].

### 4.1. Data Acquisition and Processing

We constructed our custom Python pipeline, run in the free web-based interactive computing platform Jupyter Notebooks, to automate the AT/GC data analyses. The Jupyter Notebook is the original web application for creating and sharing computational documents. It offers a simple, streamlined, document-centric environment (more details on jupyter.org). The computer code performing the here presented analyses is available on GitHub at github.com/martavohnoutova.

### 4.2. Data Treatment and Structure

We downloaded the three sequence datasets (DNA, cDNA and cds) for each species and calculated its size and GC% globally per batch and separately for each sequence in the case of cDNA and cds. These data are stored as JSON output files of two types for each species and DNA fraction. Namely, “large JSONs” contain AT/GC% and the sum of “N” separately for each sequence in all three DNA fractions. Data on the GC% of cDNA and cds individual sequences were used for histograms showing GC% the distribution for each species (not shown) to validate our results.

The species analyzed in this study are provided in the tables in Appendix A and online in the case of too large datasets, visualizing the GC% values for the three fractions in alphabetical order.

Datasets, prior to their analyses, were manually curated for redundant congeners in some model species to avoid the bias potentially introduced by the higher sequencing effort in rodents, primates, and farm animals.

### 4.3. 3D Visualization of GC% Data

The GC2C application is implemented in the Java programming language and the OpenGL graphics library (lwjgl). The GC2C visualizes individual species rendered as a small sphere and placed in 3D space. The position of the 3D sphere is based on a vector composed of GC% values of each genome fraction, i.e., DNA, cDNA and, cds. These values are normalized and converted to the interval from 0 to 1 in a unit cube with red-green-blue axes. The GC2C application is freely available on GitHub: https://github.com/fvbj/genomeVis.

## 5. Conclusions

This study elucidates that fish genomes are not as GC-poor as thought before. In contrast, in the immense diversity of fish species, highly GC-rich fish genomes exist that are even GC-richer than mammalian and avian genomes. Regarding the potential technical issues and biases, this study shows that the GC biology of vertebrates is still far from being properly understood.

For the next versions of our GC2C visualization tool, with the rate of increase in the number of species available and the increasing coverage of lineages across both vertebrates and invertebrates, a split into vertebrate orders and invertebrate phyla or other groups is foreseen. This is crucial because lineage-specific trends in GC evolution exist (Andreas et al., *in prep*). In parallel, basic information on ecology should also be involved since differences in genome evolution mostly mediated by TEs have been reported from different environments (e.g., TE-driven intron gain in aquatic eukaryotes [23], a relationship between migratory behavior and the quantitative difference reported for short interspersed nuclear (retro) elements [49], TE-driven GC enrichment in salmonid fish [50], and the GC% in marine and freshwater fish related to metabolic rate [51]).

## Figures and Tables

**Table 1:** Rounded mean GC% for each fraction in animal groups visualized in Figure 1 are ordered according to their phylogeny (first three columns). Differences between the mean GC% of the three main genome fractions (Delta 1–3, last three columns). One lancelet species is added.

Group	DNA GC%	cDNA GC%	cds GC%	Delta 1 cDNA-DNA	Delta 2 cds-cDNA	Delta 3 cds-DNA
Invertebrates	36.1	42.5	44.1	6.4	1.6	8
Tunicates	36.0	43.0	44.2	7	1.2	8.2
Lancelet	41.5	47.5	53.0	6	5.5	11.5
Cyclostomes *	46.3	53.0	54.9	6.7	1.9	8.6
Shark	42.3	47.2	48.3	4.9	1.1	6
Bichir	39.3	44.5	45.6	5.2	1.1	6.3
Coelacanth	41.1	44.4	46.5	3.3	2.1	5.4
Fish	40.9	49.4	52.5	8.5	3.1	11.6
Amphibians	41.9	45.9	46.8	4	0.9	4.9
Reptiles	43.1	48.1	48.7	5	0.6	5.6
Birds	41.7	49.7	50.1	8	0.4	8.4
Mammals	41.1	50.9	51.9	9.8	1	10.8

* Cyclostomes are a monophyletic group comprising lampreys and hagfishes [29].

**Table 2:** Manually curated minimal and maximal GC% of DNA from Ensembl and NCBI.

Group	Min DNA GC%	Max DNA GC%
Invertebrates	*Pediculus humanus* ~28%	*Anopheles gambiae* ~44%
Cephalochordates	*Asymmetron lucayanum* ~40%	*Branchiostoma floridae* ~42%
		Tetraodontidae ~44–46%
Fish	*Danio rerio* ~36% *	*Thaleichthys pacificus* ~46%
		*Alosa alosa* ~48%
Amphibians	*Limnodynastes dumerilii* ~37%	*Ambystoma mexicanum* ~45%
Reptiles	*Notechis scutatus* ~37.2%	*Sphaerodactylus townsendi* ~46%
Birds	*Poecile atricapillus* ~40%	*Pogoniulus pusillus* ~46%
Mammals	*Sarcophilus harrisii* ~37–38% *Monodelphis domestica* ~38%	*Ochotona princeps* ~44% *Ornithorhynchus anatinus* ~46%

* Zebrafish is the teleost species with a high-quality chromosome level genome assembly and the lowest GC% (lower GC% has been recorded in several other cypriniform species, however, with a lower-quality scaffold or contig level genome assembly).

## Data Availability

Data and codes supporting reported results can be found can be found in following GitHub repositories: https://github.com/fvbj/genomeVis for the 3D GC2C visualization tool and https://github.com/martavohnoutova for codes used to analyze sequence data in Jupyter Notebooks and the generated results.

## Appendix A

### Specific Details on Proportions of cDNA and cds in Animal Genomes

Within vertebrates, fish show the highest proportions as well as the highest IQR also regarding their sample size (N). The only two amphibians available show an intermediate IQR between 5 and 10% for cDNA and approximately 5% for cds. Beginning with reptiles (N = 12), a decrease in both the IQR and the size of these two fractions is apparent and persists in birds and mammals. In mammals and birds, the narrow IQR exists despite their (er) high sample size N. Invertebrates as an outgroup to chordates similarly as fish show larger proportions of cDNA (2.4–58.5%) and cds (1.6–42%) resulting in a high IQR with a comparable N to fish. The only two species of tunicates (both sea squirts of the genus *Ciona*) show an even higher averaged proportion of both cDNA (18.5%) and cds (16.4%) than invertebrates. Whereas both cyclostomes, the sea lamprey, and the inshore hagfish, show a low proportion and a narrow IQR for cDNA (2%) and cds (1.5%), respectively, despite their high phylogenetic distance. In the single shark species available in Ensemble, the elephant shark, cDNA occupies approx. 12% and cds 10% of its genome. Due to its unusually large genome, a single bichir species, the reedfish, is shown to have low proportions of cDNA (2%) and cds (1.6%).
